# Indications, Efficacy, Safety, and Clinical Outcomes of 585 nm Pulsed Dye Laser in Non-Malignant Laryngeal Lesions: A Systematic Review

**DOI:** 10.3390/jpm13091374

**Published:** 2023-09-14

**Authors:** Henar González-Rodríguez, Miguel Mayo-Yáñez, Alberto Maria-Saibene, Fabiana Allevi, Carlos M. Chiesa-Estomba, Luigi A. Vaira, Jerome R. Lechien

**Affiliations:** 1 Otorhinolaryngology—Head and Neck Surgery Department, Complexo Hospitalario Universitario A Coruña (CHUAC), 15006 A Coruña, Spain; 2 Young-Otolaryngologists of the International Federation of Oto-Rhino-Laryngological Societies (YO-IFOS), Research Study Group, 75000 Paris, France; alberto.saibene@gmail.com (A.M.-S.); fabiana.allevi@gmail.com (F.A.); chiesaestomba86@gmail.com (C.M.C.-E.); luigi.vaira@gmail.com (L.A.V.); jerome.lechien@umons.ac.be (J.R.L.); 3 Otolaryngology Unit, Santi Paolo e Carlo Hospital, Department of Health Sciences, Università degli Studi di Milano, 20142 Milan, Italy; 4 Maxillofacial Surgery Unit, Santi Paolo e Carlo Hospital, Department of Health Sciences, Università degli Studi di Milano, 20142 Milan, Italy; 5 Otorhinolaryngology—Head and Neck Surgery Department, Hospital Universitario Donostia—Biodonostia Research Institute, 20014 Donostia, Spain; 6 Maxillofacial Surgery Operative Unit, Department of Medicine, Surgery and Pharmacy, University of Sassari, 07100 Sassari, Italy; 7 Department of Otolaryngology, Polyclinique de Poitiers, Elsan Hospital, 86000 Poitiers, France; 8 Department of Otolaryngology—Head & Neck Surgery, Foch Hospital, School of Medicine, UFR Simone Veil, Université Versailles Saint-Quentin-en-Yvelines (Paris Saclay University), 91190 Paris, France; 9 Department of Human Anatomy and Experimental Oncology, UMONS Research Institute for Health Sciences and Technology, University of Mons (UMons), 7000 Mons, Belgium; 10 Department of Otolaryngology—Head & Neck Surgery, CHU Saint-Pierre (CHU de Bruxelles), 1000 Brussels, Belgium

**Keywords:** 585 nm pulsed dye, laser, head neck, otolaryngology, surgery, lesion, vocal, laryngeal, larynx, voice

## Abstract

The objective of this manuscript was to review the indications, efficacy, and safety of a 585 nm pulsed dye laser (PDL) in non-malignant laryngeal lesions. Following the PRISMA statement recommendations, three independent authors searched for articles published in PubMed/MEDLINE, the Cochrane Library, Google Scholar, Scielo, and Web of Science. A bias analysis was performed following NICE guidance tools. From the 506 identified publications, 19 observational studies met the inclusion criteria. The PDL improves vocal quality objectively and subjectively in vascular lesions (*p* < 0.005) and improves vocal quality in patients with dysplasia/leukoplasia without changing the natural history of the disease compared to other treatments. Reinke’s edema and granulomas require an average of 1.5 PDL sessions for resolution. Treatment of recurrent respiratory papillomatosis requires multiple sessions, with complete remission achieved in 50–70% of patients. Regardless of the lesion, the tolerance of the procedure under local anesthesia is exceptional (84–97%), and the results in terms of regression and vocal quality are promising. The complication rate is minimal, and the procedure does not interfere with other treatment alternatives. There is no consensus on laser settings. The lack of consistent use in evaluating vocal outcomes, whether objective or subjective, prevents the comparability between studies. The 585 nm pulsed dye laser appears to be an effective and safe therapeutic option in patients with non-malignant laryngeal pathology. Future controlled studies are needed to compare the 585 nm pulsed dye laser with other lasers or cold instrument procedures.

## 1. Introduction

The CO_2_ laser has historically been the most widely used for treating various laryngeal lesions [1]. However, with technological advancements, the pulsed photoangiolytic laser has gained prominence due to its capacity to preserve the epithelial layer, leading to lesion regression, improved clinical and vocal symptoms, and a low rate of complications [2]. The 585 nm pulsed dye laser (PDL) is a photoangiolytic laser widely used for dermatological purposes since the 1980s. Its wavelength aligns with the peak absorption of hemoglobin, its intended target. As a result, it can coagulate the microcirculation beneath the lesion with minimal thermal damage to the surrounding tissue [3]. This selectivity in energy absorption via hemoglobin may result in less damage and scarring to the vocal cords compared to other lasers commonly used in laryngeal surgery. After its introduction into laryngeal surgery in the 1990s [4], the PDL has been the subject of limited studies, revealing new applications in laryngeal surgery, particularly for in-office procedures. The current body of literature does not yet provide a consensus on the indications for PDL use, its safety and efficacy profile, or clinical outcomes for office-based and inpatient suspension microlaryngoscopy PDL procedures. Therefore, the objective of this systematic review was to investigate the existing literature on the indications, efficacy, and safety of PDL procedures.

## 2. Materials and Methods

The review followed the guidelines outlined in the Preferred Reporting Items for a Systematic Review and Meta-analysis (PRISMA) [5]. To structure the review process, a framework based on Population, Intervention, Comparison, Outcome, Timing, and Setting (PICOTS) was employed [6].

### 2.1. Participants and Inclusion/Exclusion Criteria

We included human-published studies from English or Spanish peer-reviewed journals that presented data on patients with clear descriptions of non-malignant laryngeal lesions treated using the 585 nm PDL procedure and provided details about the methods used for lesion diagnosis. We did not consider preprint studies, grey literature, reviews, pre-clinical studies, or conference communications. Eligibility criteria related to the type of study (both experimental and observational, prospective and retrospective) were not applied. The included studies had to establish the diagnosis of vocal fold lesions through videolaryngostroboscopy or histological examination. The surgical procedure could be conducted in-office (under local anesthesia) or with in-suspension microlaryngoscopy (under general anesthesia).

### 2.2. Intervention and Comparison

The primary intervention of studies was the microsurgery of vocal folds through a 585 nm PDL. The PDL procedure was the only procedure type in studies or was compared with the following: cold instrument microsurgery, CO_2_, KTP, or TrueBlue laser microsurgeries. In the case of several groups, the comparison of clinical and therapeutic outcomes was considered. 

### 2.3. Outcomes

The primary outcome of this review was to assess the effectiveness of 585 nm PDL therapy (disease control, resolution, and/or pre- to post-procedure voice quality), including the feasibility of the procedure for the underlying lesion (complications, patient tolerance, and pain management). The main data collected were the publication information (year, country, and study design), demographic information (number, age, and gender of patients), 585 nm PDL therapy parameters, follow-up information, and the existence and features of the control group.

### 2.4. Search Strategy

**Figure 1:**
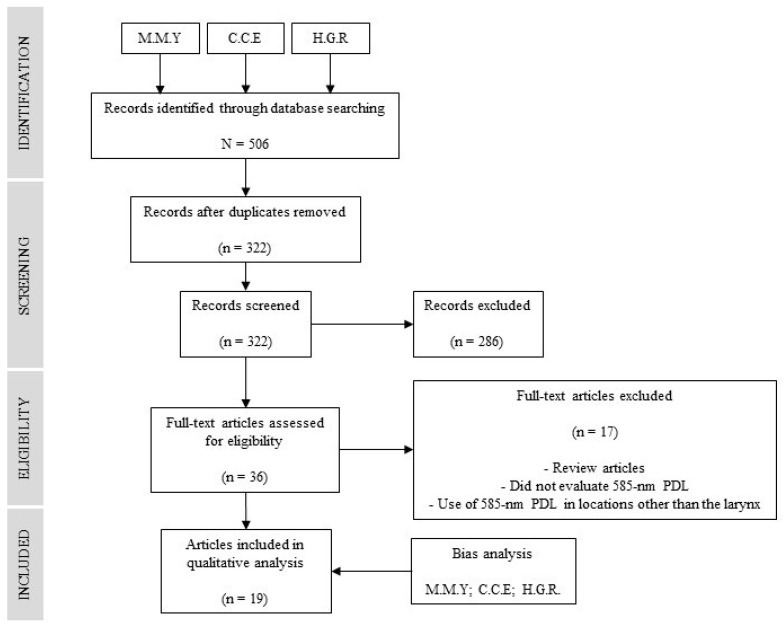
PRISMA statement flow diagram.

Three independent authors (MMY, HGR, and CCE) conducted a comprehensive literature search using various databases, including PubMed/MEDLINE, the Cochrane Library, Google Scholar, Scielo, and Web of Science. Additionally, a meta-search was performed using the Trip Database. The search was conducted using a set of keywords (and/or) that included: “585 nm pulse dye”, “laser”, “surgery”, “lesion”, “vocal”, “laryngeal”, “larynx”, and “voice”. The authors initially screened abstracts and available full-text publications related to the condition of interest. After removing duplicate articles and selecting relevant ones, the authors proceeded to read the complete texts of these selected articles and reviewed their bibliographic references. This thorough process aimed to ensure the inclusion of any potentially relevant studies that might not have been identified through the initial search strategy. In cases where disagreements arose among the authors, these were resolved through discussion within the work team, ultimately making consensus-based decisions (Figure 1).

### 2.5. Data Analysis

Data extraction was conducted in duplicate to minimize errors in the qualitative analysis. When publications originated from the same research center, potentially containing duplicate samples, all relevant publications were included in the qualitative analysis. However, for the quantitative analysis, only the study with the largest sample size was considered. The level of evidence was categorized following the guidelines of the Oxford Centre for Evidence-Based Medicine Levels [7]. The methodological quality of the chosen studies was assessed using the National Institute for Health and Care Excellence (NICE) Public Health Guidance tool [8]. Due to the heterogeneity of the data and the limited sample size, it was not feasible to conduct a statistical analysis.

## 3. Results

A total of 506 articles were identified. Once duplicates and unrelated items were excluded, 39 papers were selected. Their full text was evaluated according to the proposed methodology, after which 17 manuscripts were excluded because they were reviews [9,10,11], in vitro studies [2,12], did not evaluate the PDL 585 nm [13,14,15,16,17,18,19,20], or they used the PDL 585 nm in locations other than the larynx [21,22,23]. The final result included 19 articles: 7 where the procedure was performed in the operation room under general anesthesia (Supplementary Table S1), and 12 where the procedure was performed in-office under local anesthesia (Supplementary Table S2).

### 3.1. Vascular Lesions (Varices, Ectasias, and Polyps)

Zeitels et al. compared the results of the 585-PDL and 532-KTP laser procedures under general anesthesia in professional singers and found no differences [24]. No patient had an episode of post-surgical vocal hemorrhage. All subjects returned to active singing without a subjective report of vocal impairment and without changes in videostroboscopy or acoustic measurements. Substantially more superficial lamina propria (SLP) ecchymosis due to vessel wall rupture was observed in the PDL group, and the onset of singing activities was delayed from 4 to 5 weeks. Irradiation of larger varicose veins and ectasias with PDL tended to cause vessel wall rupture and visible extravasation of blood in the SLP. Failing to drain the blood could have led to fibrosis and scarring of the delicate mucosa of the vocal fold if the laser was further used. These findings maintain a preference for the KTP laser over PDL, based on ensuring adequate hemostasis and avoiding the risk of vascular rupture, thus avoiding a subsequent cordotomy for the drainage of subepithelial hemorrhage. They conclude that PDL treatment, despite lengthening the recovery time, also allows an optimal final result to be achieved.

Subsequent studies were performed under local anesthesia without complications [25,26,27,28]. Kim et al. [26] obtained a regression of the lesion in all patients, with a significant decrease in the GRBAS scale after operation (*p* < 0.001). The HNR (noise-to-harmonic ratio) increased, and jitter, shimmer, sPPQ (smoothed pitch perturbation quotient), sAPQ (smoothed amplitude perturbation quotient), and SPI (soft phonation index) decreased after surgery (*p* < 0.05). These results contrast those obtained by Ivey et al. [27], in which only 38% of cases had greater than 70% improvement, more than one intervention was necessary, and the response to the treatment was size-dependent.

### 3.2. Keratosis/Leukoplakia and Dysplasia

A total of two studies involving procedures under general anesthesia [29,30] and seven with in-office procedures [25,28,31,32,33,34,35] were found. Park et al. [29] conducted a comparative study between the CO_2_ laser and the PDL, demonstrating that the PDL improves the voice outcome compared to the CO_2_ laser in treating vocal cord leucoplakia (jitter and VHI). No differences were found in recurrence or the need for repeating treatment, which was necessary in four patients treated with the PDL. In this regard, Ayala et al. obtained a complete resolution in all patients, although repeating the treatment was necessary for two patients [30]. Similar results were obtained by other authors, with a VHI decrease from 10 to 5 (IQR: 4.3–16.3, *p* = 0.037) [31] or a mean VHI improved from a preoperative value of 21 ± 10 to a postoperative value of 14 ± 11 (*p* < 0.001) [28].

Laryngeal leucoplakia and dysplasia are defined by local recurrence and the need for repeated treatment procedures. The possibility of an in-office procedure could help patients to avoid repeated general anesthesia [33,34]. Centric et al. [25] demonstrated good tolerance and safety of the in-office procedure, possible in 97% of their patients. Koss et al. [31] observed that serial in-office treatment with the PDL appears effective for disease control with minimal morbidity, preservation of voice quality, and achieving prolonged periods of non-treatment. This statement is contrary to that reported by Zeitels et al., in which they did not observe that the PDL alters the natural history of recurrence of dysplasia [35].

Given the characteristics of the PDL, it appears to be the ideal laser for treating leucoplakia and dysplasia in the anterior commissure due to its low rate of synechiae formation [28]. On the other hand, for treating large lesions or if hemostasis is a concern, the best choice is using CO_2_ or Tm:YAG lasers [32]. Special mention should be made of the phototherapeutic combination of the PDL with ALA (aminolevulinic acid) [31,33]. When cells absorb the ALA, there is a natural accumulation of the photosensitive protoporphyrin IX (PpIX). Although all cells that absorb the ALA will convert it into PpIX, it is believed that diseased cells will more quickly accumulate PpIX and make them more susceptible to damage from phototherapy. Unfortunately, to date, there are no other studies evaluating its efficacy in the larynx.

### 3.3. Reinke’s Edema

Only two studies used PDL therapy to treat Reinke’s edema; both were in-office under local anesthesia [28,32]. Koufman et al. performed 18 PDL procedures in 12 patients (mean 1.5, range 1–3). For 10 patients, follow-up was available (mean 7.3 months, range 1–13 months). Two patients had small vocal fold hemorrhages that were ultimately resolved. All patients experienced complete resolution with subjective voice quality improvement and normal videostroboscopy [32]. Similar results were found by Mouadeb et al., requiring 16 procedures in 10 patients, where two patients were under general anesthesia (20%). One patient with Reinke’s edema developed a stridor after the procedure, requiring a 3-day hospital admission for observation and intravenous corticosteroids. There was no incidence of vocal cord webbing or scarring, and no other complications occurred [28]. Vocal analysis was not reported in any of the studies.

### 3.4. Granulomas and Scars

Six studies assessed the utility of the PDL for treating granulomas that are unresponsive to anti-reflux treatment [25,28,32,34,36,37]. Centric et al. evaluated the utility and safety of the in-office PDL procedure for vocal fold granulomas. They demonstrated that the procedure was safe, but all patients treated required a combination of in-office and operating room procedures [25]. No data on voice analysis were reported. Along the same lines are the results found by Koufman et al., with a mean of 1.6 procedures/patient (range 1–5) in treating granulomas and a 68% effectiveness. Six subjects needed further surgery under general anesthesia [32]. These results of partial improvement were also reported by Clyne et al. (50% effectiveness) [36]. The PDL in the treatment of granulomas is a safe procedure, with 84% of patients requiring no analgesia [34] and only one complication reported concerning a PDL fiber tip rupture in the trachea (immediately retrieved with cup forceps) [32].

Mortensen et al. concluded that vocal scar treatment with a PDL improved the VHI from 48.44 to 35.55 (*p* < 0.05) at 6 months post-treatment. The same happened with the VHI functional, emotional, and physiological (*p* < 0.05) results. Similarly, the mean phonatory flow significantly increased from 0.177 to 0.254 L/S (*p* < 0.05). The remaining parameters did not report statistically significant differences [37].

### 3.5. Recurrent Respiratory Papillomatosis (RRP)

Together with leukoplakia, laryngeal papillomatosis was the pathology with the most studies performed, with four in which the procedure was performed under general anesthesia [4,38,39,40] and five in-office [25,28,32,34,35]. The term RRP is defined by instances of repeated local recurrence and the necessity for recurrent treatment, which makes the in-office procedure an intriguing option to assist patients in avoiding repeated general anesthesia.

Several authors point out the good results obtained with the PDL, without damage to the epithelium and allowing opposing surfaces to be treated simultaneously with no evidence of scarring or web formation [28,32,38,39]. Despite this, PDL papilloma treatment is not curative; a true disease-free interval can only be assessed with continued surveillance. There is no evidence to expect that recurrence rates will differ between the PDL and standard CO_2_ laser surgery, and some authors point out that bulky nonvascular lesions such as exophytic papillomas or supraglottic cysts are typically best excised or ablated using either the CO_2_ laser or the Tm:YAG laser [32]. Valdez et al. obtained a complete resolution in seven patients, two partial (>50%) regressions, and one lost follow-up [40]. Similar results in terms of resolution were obtained by other authors [30,39]. As for the in-office studies, Koufman et al. required a mean of 3.6 procedures/patient (range 1–15) to obtain control of the disease. In a 15% (9/59) of the cases, the completion of successive procedures in the operating room was necessary [32]. None of the studies had voice outcomes analysis. 

### 3.6. Evidence Level and Risk of Bias 

The level of evidence of all the studies included in this review is low (level 4), obtaining a 52.63% “good quality” score in the bias analysis (Table 1). Among the studies included, 16 were uncontrolled studies (9 prospective [4,27,30,33,35,37,38,39,40] and 7 retrospective [25,26,28,31,32,34,36]), and 1 was a case report [41]. A prospective controlled study was found (Supplementary Table S1) [24], but the authors did not compare the outcomes between the PDL and KTP lasers. Finally, a retrospective controlled study (Supplementary Table S1) evaluated the PDL and CO_2_ lasers [29]. Lastly, a retrospective controlled study (refer to Supplementary Table S1) assessed the use of the PDL and CO_2_ lasers [29], yet the authors did not compare the outcomes.

The laser settings were totally or partially specified in most studies (Table 1 and Table 2). In four of the studies, the laser settings were not specified [25,34,38,41]. There are variations in the settings for the same diseases across different studies (Supplementary Tables S1 and S2) and between different pathologies [32]. Eleven studies evaluated voice outcomes [24,26,27,28,29,31,32,35,37,38,41], presenting a significant heterogeneity in data collection (Table 2). In 15 studies, PDL-associated complications were reported [4,24,25,26,27,28,29,30,32,33,35,36,38,39,40]. In 15 studies, disease control was reported [4,25,26,27,28,30,31,32,33,35,36,37,39,40,41]. Only one study evaluated the pain and comfort of in-office procedures [34].

## 4. Discussion

The 585 nm PDL was introduced into laryngology as a potential fiber-based laser photoangiolytic treatment, offering the possibility of in-office procedures as well as during suspension microlaryngoscopy, with few postoperative complications and favorable outcomes in terms of voice quality. This review highlights that over the past decade, there has been a growing number of reports on the use of PDL in various benign laryngeal conditions. Currently, the PDL is used for several indications, and it has shown positive results. The PDL is currently used for several indications, with favorable results, although there are several competitors, such as the KTP laser [43], other lasers with similar wavelengths [44], or the more recent CO_2_ fiber laser [45].

The fact that the PDL (and all office-based photoangiolytic lasers) is available for in-office use greatly increases its popularity, as office-based procedures offer decreased cost and procedure length. Rees et al. [42] calculated that USD 5000 was saved per case when moving a procedure from the operating room to the office. Performing these procedures in-office significantly reduces the time of a patient’s presence at the hospital by reducing preoperative, procedure, and recovery time compared with the operation room [46]. The lack of general anesthesia, the safety, and the ease of scheduling in-office procedures are all cost-effective arguments for developing local procedures in laryngology. 

Due to the characteristics of the PDL, it can act through two mechanisms: selective thermolysis of the subepithelial microvasculature and creation of a cleavage plane between the basal epithelial cells and the SLP, with changes observed within the lamina lucida layer of the basement membrane zone due to the denaturation of anchoring proteins [2]. For this reason, vascular lesions in the larynx represent the ideal target for the PDL, essentially absorbed by oxyhemoglobin. Typical of voice professionals, due to overuse, mechanical vocal trauma and/or chronic inflammation, varicose veins, and vascular ectasias occur in the microvasculature at the level of the lamina propria. They tend to rupture and bleed, representing the usual indication for treatment [24]. The combination of its photocoagulation properties and its selective anxiolytic effect leads to the disappearance of the lesion over time. In this sense, the 585-PDL is less absorbed by oxyhemoglobin than the 532-KTP, although studies suggest that this is not clinically significant, and selective optical penetration/absorption into blood vessels and surrounding tissues is similar for each laser [31,47]. Zeitels et al. found differences between the two lasers, with a higher frequency of vessel wall rupture with the 585 nm PDL, leading to a delayed resumption of vocal activities, although it did not impair the final success of the treatment [24]. These differences have not been without debate [32,48,49].

Regarding the limitations of the technique, especially at the in-office level, the articles included point out the need to standardize this procedure. Among the aspects to be considered are the laser power required and the duration of the light pulse. The first issue is closely related to the location and characteristics of the lesion, which determines the angulation and distance between the tip of the laser fiber and the tissue to be treated; the maximum incidence corresponds to a perpendicular angulation. Regarding the second point, the width of the light pulse (msec) should be proportional to the thermal relaxation time of the microvasculature, which, in turn, is in accordance with the vascular diameter. In case of disparity, a suboptimal result and/or injury of adjacent tissues will be achieved. Clinical efficacy must be balanced against secondary thermal damage [1]. Therefore, it is useful to use the laser in pulses of light as this results in a shorter exposure time. In this sense, an important distinguishing characteristic between the PDL and KTP lasers is that the KTP is a solid-state laser, and its pulse width can be varied substantially more than that of the PDL. This allows for the delivery of pulsed laser energy over a time period that can be up to 30 times longer for the KTP than for the PDL [2]. Despite these theoretical differences between anxiolytics and cutting/ablating lasers, to date, no randomized study in benign laryngeal pathology exists.

The primary limitation of the present review is the low number of included studies and the low number of patients in studies. The lack of randomized controlled studies comparing different laser procedures in patients with the same clinical profile is an additional weakness of the current literature, which limits the quality of any review. Additionally, regarding vocal outcomes, the inconsistent utilization of objective and subjective assessment tools for evaluating vocal outcomes complicates the ability to compare results across studies and ultimately hinders the potential for further meta-analytical insights. Finally, it should be noted that most studies on PDLs are 10 years old or more, which could indicate a loss of research interest in PDLs compared to other options such as the KTP.

## 5. Conclusions

Technology has expanded our options in laser surgery, and choosing the appropriate laser for a given procedure should be performed on a case-by-case basis. An in-office PDL seems to be a safe procedure and may be proposed for selected patients with benign lesions of the vocal folds, including unilateral or bilateral Reinke’s edema, cyst, vascular lesions, vocal scars, ectasia, polyps, papillomatosis, dysplasia, leukoplakia, and granuloma. For certain medical conditions, office-based treatment may be a one-time event. However, in cases like leukoplakia and papilloma, the disease tends to recur, necessitating repeated treatments. This underscores the significance of considering office-based care as a viable option. Nevertheless, the recent literature on angiolytic lasers has primarily concentrated on other lasers that are more readily accessible, such as the KTP or TruBLue lasers, despite their similar wavelengths.

## Figures and Tables

**Table 1:** Risk bias evaluation.

	Reference	Evidence Level	#1	#2	#3	#4	#5	#6	#7	#8	#9	Quality Rating (Good, Fair, or Poor)
1.	Koss et al. [31]	4	Yes	Yes	No	Yes	Yes	Yes	Yes	Yes	Yes	Good
2.	Ihenachor et al. [41]	4	Yes	No	NA	NA	No	Yes	CD	NA	Yes	Poor
3	Park et al. [29]	4	Yes	Yes	Yes	Yes	No	Yes	Yes	Yes	Yes	Good
4.	Centric et al. [25]	4	Yes	Yes	Yes	Yes	No	No	CD	No	No	Poor
5.	Kim et al. [26]	4	Yes	No	CD	Yes	Yes	Yes	Yes	Yes	Yes	Good
6.	Mortensen et al. [37]	4	Yes	Yes	Yes	Yes	Yes	Yes	Yes	Yes	Yes	Good
7.	Koufman et al. [32]	4	Yes	Yes	Yes	Yes	Yes	No	Yes	No	Yes	Good
8.	Ivey et al. [27]	4	Yes	No	Yes	Yes	Yes	Yes	Yes	No	Yes	Good
9.	Hartnick et al. [38]	4	Yes	No	CD	CD	No	No	CD	No	No	Poor
10.	Zeitels et al. [24]	4	Yes	No	CD	Yes	Yes	Yes	CD	No	No	Poor
11.	Franco [33]	4	Yes	No	Yes	Yes	Yes	Yes	Yes	No	Yes	Good
12.	Mouadeb et al. [28]	4	Yes	Yes	CD	No	Yes	Yes	Yes	No	Yes	Good
13.	Rees et al. [34]	4	Yes	No	CD	CD	No	Yes	CD	No	Yes	Poor
14.	Clyne et al. [36]	4	Yes	No	Yes	Yes	Yes	No	Yes	No	Yes	Fair
15.	Zeitels et al. [35]	4	Yes	No	Yes	Yes	Yes	Yes	CD	No	Yes	Good
16.	Ayala et al. [30]	4	Yes	Yes	Yes	Yes	Yes	Yes	Yes	No	Yes	Good
17.	Franco et al. [39]	4	Yes	No	Yes	CD	Yes	No	CD	No	No	Poor
18.	Valdez et al. [40]	4	Yes	Yes	CD	No	Yes	No	No	No	Yes	Poor
19.	McMillan et al. [4]	4	Yes	Yes	CD	Yes	Yes	No	No	NA	Yes	Fair

CD, cannot determine; NA, not applicable; NR, not reported. #1. Was the study question or objective clearly stated? #2. Was the study population clearly and fully described, including a case definition? #3. Were the cases consecutive? #4. Were the subjects comparable? #5. Was the intervention clearly described? #6. Were the outcome measures clearly defined, valid, reliable, and implemented consistently across all study participants? #7. Was the length of follow-up adequate? #8. Were the statistical methods well-described? #9. Were the results well-described?

**Table 2:** Voice quality outcomes.

Voice Quality Evaluations	References
None	[4,25,30,33,36,39,40,42]
Subjective	
GRBAS scale	[26]
Self-assessment	[35,37,41]
VHI or VHI-10	[28,29,31,37]
CAPE-V	[24]
VLS evaluation (wave, etc.)	[24,26,27,29,32,37,38]
Objective	
Acoustic	
Jitter	[24,26,29,37]
Shimmer	[26,29,37]
HNR/NHR	[24,26,29,37]
F0 or range F0 or MF0	[24,29]
sPPQ, sAPQ or SPI	[26]
QxM, CFx, or CAx	[29]
Aerodynamic	
Maximum phonation time	[29]
Oral sound pressure level/subglottal air pressure	[24]
Sound pressure level	[24]
Mean airflow rate	[29,37]

Abbreviations: F0, fundamental frequency; GRBAS, grade, roughness, breathiness, asthenia, and strain; HNR, harmonic-to-noise ratio; QxM, mean closed quotient; CFx, percentage irregularity of frequency; CAx, percentage irregularity of amplitude; NHR, noise-to-harmonic ratio; sPPQ, smoothed pitch perturbation quotient; sAPQ, smoothed amplitude perturbation quotient; SPI, soft phonation index; VHI, voice handicap index; VLS, videolaryngostroboscopy.

## Data Availability

Not applicable.

## Supplementary Materials

The following supporting information can be downloaded at https://www.mdpi.com/article/10.3390/jpm13091374/s1, Table S1: Characteristics of studies with procedure under general anesthesia. Table S2: Characteristics of studies with procedure under local anesthesia.
