# Supplementary material for: Indications, Efficacy, Safety, and Clinical Outcomes of 585 nm Pulsed Dye Laser in Non-Malignant Laryngeal Lesions: A Systematic Review
Source: J Pers Med. 2023 Sep 14;13(9):1374. doi: 10.3390/jpm13091374 (PMC10533112; doi:10.3390/jpm13091374)
Supplement: Supplementary file 1 [file jpm-13-01374-s001.zip › jpm-2588771-supplementary.pdf]

SUPPLEMENTARY TABLES:

Supplementary Table S1. Characteristics of studies with procedure under general anesthesia.

Authors (Country, year)	Study	Characteristics	Lesions	Laser settings	Outcomes	Results	Findings
Park et al.[29]<br>(Republic of Korea, 2014)	Retrospective controlled (2007-2012)	PDL:<br>- N/Proc: 19/>19<br>- Age: 57.6<br>- Gender: 19/0<br>CO2:<br>- N/Proc: 17/>17<br>- Age: 59.5<br>- Gender: 17/0	Leukoplakia	50–70 pulses<br>Output of 600 mJ/pulse (range, 100–700 mJ per pulse)<br>1 Hz repetition rate	Complications<br>Voice outcome	Subjective improvement of voice was observed in both patient groups.<br>Only the jitter parameter was shown to be significantly decreased in PDL vocal cord mucosectomy.<br>A diminution or lack of mucosal wave was observed in more CO2 laser vocal cord treatment group cases.<br>Mean follow-up 29.6 mo.<br>No significant complications.	PDL, compared to CO2 laser in the treatment of vocal cord leukoplakia, improves the voice outcome.
Hartnick et al.[38]	Observational uncontrolled	N/Proc: 23/37	RRP	NA	Complications	The epithelium is not damaged by PDL therapy.	Postoperative RRP staging scores and

(USA, 2007)		Age: 6-18 mo<br><br>Gender: 15/8				Opposing surfaces can be treated simultaneously with no evidence of scarring or web formation.	formal voice outcomes were not evaluated.
Zeitels et al.[24]<br><br>(USA, 2006)	Prospective controlled (2001-2006)	PDL:<br><br>- N/Proc: 24/33<br>- Age: NA<br>- Gender: NA<br><br>KTP:<br><br>- N/Proc: 15/21<br>- Age: NA<br>- Gender: NA	Varices and ectasias	0.45 ms pulse width<br><br>5 J per pulse maximum output<br><br>2 Hz repetition rate<br><br>0.6 mm fiber, 1 to 2 mm spot size, approximately 20 to 80 J/cm2 fluence.	Complications<br><br>Voice outcome	More ecchymosis of the SLP from vessel wall rupture in the PDL group.<br><br>No patient has had a postsurgical episode of vocal hemorrhage.<br><br>All subjects returned to active singing without the subjective report of vocal deterioration.	Microcirculatory vessel wall rupture occurred frequently with the 585 nm PDL.<br><br>It delayed the resumption of vocal activities, although it did not impair the final success of the treatment.<br><br>The 532 nm pulsed KTP laser was more effective and easier to use than the 585 nm PDL for involuting ectasias and varices

Ayala et al.[30]<br>(USA, 2005)	Prospective uncontrolled (1999-2001)	N/Proc: 9/11<br>Age: 55<br>Gender: 5/4	Keratosis	450 μs pulse width<br>2.2 J/pulse maximum output<br>2 Hz repetition rate<br>0.6 mm fiber	Complications<br>Disease Control<br>Ultrastructural examination	Complete resolution.<br>Two recurrences with complete resolution after repeat PDL.	The PDL creates a cleavage plane between the basal epithelial cells and the SLP, with changes observed within the lamina lucida layer of the BMZ.
Franco et al.[39]<br>(USA, 2002)	Prospective uncontrolled (1999-2001)	N/Proc: 23/41<br>Age: NA<br>Gender: 12/11	RRP	450 μs pulse width<br>5 J/pulse maximum output<br>1 Hz repetition rate.<br>1 mm fiber, 1 to 2 mm spot size<br>38 to 255 J/cm^2 fluence	Complications<br>Disease Control	1.78±0.74 surgeries (range 1-3).<br>Photocoagulation of the microvasculature of both vocal folds was obtained without resection in 26 (70%) of bilateral disease.<br>Minimal bleeding in 13 cases in which epithelium was removed after PDL therapy.<br>No complications.	Voice quality assessments and follow-up time not provided.<br>585 nm PDL was effective in enhancing the eradication of disease without the clinically observed soft tissue complications associated with the CO2 laser.<br>PDL treatment of papilloma is not

							curative; true disease-free interval can only be assessed with continued surveillance.
Valdez et al.[40]<br><br>(USA, 2001)	Prospective uncontrolled	N/Proc: 10/16 (2 of them in-office)<br><br>Age: 34.2<br><br>Gender: 8/2	RRP	300-500 µs duration.<br><br>Fluences 6-10 J/cm^2 with 1 to 5 pulses per irradiation site.<br><br>1 mm diameter optical fiber. 2.7 mm diameter spot.	Complications<br><br>Disease Control	7 complete resolution, 2 partial (>50%) regression, 1 lost follow-up.<br><br>With partial regression: 1 new PDL and 1 CO_2. 6 patients presented recurrence (mean time 7 mo).<br><br>Follow-up 1-22 mo (mean 11 mo).<br><br>No complications.	Voice quality assessments not provided. Subjective improvement of voice after treatment.<br><br>PDL causes clinical regression of vocal cord papillomas without evidence of soft tissue complications or changes in vocal cord function.<br><br>The technique does not rely on precise spatial control or extraordinary surgical

							skills to achieve satisfactory results.
McMillan et al.[4] (USA, 1998)	Prospective uncontrolled	N/Proc: 3/3 Age: 37, 25, and 45, respectively. Gender: 2/1	RRP	300-500 μs duration. 6 J/cm² (two pulses per irradiation site), 8 J/cm² (single pulses per irradiation site), and 10 J/cm² (single pulses per irradiation site). 2.7 mm diameter laser spot	Complications Disease Control	Patient 1: 1 hemilarynx with CO2 laser, 1 hemilarynx with PDL. No recurrence on both sides after 2 mo. Patient 2: CO2 and PDL combination. At 4 mo, recurrence. At 6 mo, new laser therapy. Patient 3: 1 hemilarynx with CO2 laser, 1 hemilarynx with PDL. Recurrence only in CO2-treated places. No complications.	Voice quality assessments not provided. 585 nm PDL may provide a minimally traumatic alternative to standard CO2 laser surgery. PDL treatment of papilloma is not curative; true disease-free interval can only be assessed with continued surveillance. There is no reason to expect that recurrence rates will differ between PDL and

							standard CO2 laser surgery.

Abbreviations: N/Proc, number/procedures; NA, not available; M/F, male/female; RRP, recurrent respiratory papillomatosis; mo, month(s); GRBAS, grade, roughness, breathiness, asthenia, strain; MF0, mean fundamental frequency; MPT, maximal phonation time; F0, fundamental frequency; NHR, noise-to-harmonic ratio; N/proc, number of patients/procedures; PDL, pulsed dye laser; SPL, sound pressure level; VF, vocal fold(s); VHI, voice handicap index.

Supplementary Table S2. Characteristics of studies with procedure under local anesthesia.

Authors (Country, year)	Study	Characteristics	Lesions	Laser settings	Outcomes	Results	Findings
Koss et al.[31]<br><br>(USA, 2017)	Retrospective uncontrolled (2008–2015)	100 in-office laser treatments in 46 patients.<br><br>KTP only: 40<br><br>KTP/ PDL on different visits: 5<br><br>PDL only: 1	Leukoplakia	KTP: 32.5 watts (IQR: 30–35), pulse width 15.0 ms (IQR: 15–15), 124 joules (IQR: 95.7–155)<br><br>PDL: 159.5 joules (IQR: 138–201.8)	Disease Control<br><br>Voice Outcomes	Overall disease regression using ImageJ (n=43) was median 77.1% (IQR: 42.2–100), ($P < 0.001$).<br><br>VHI decrease from 10 to 5 (IQR: 4.3–16.3, $P = 0.037$).<br><br>Median follow-up 19.6 mo.	Serial in-office KTP or PDL treatment appears to be effective for disease control with minimal morbidity and preservation of voice quality.

		8 patients received topical ALA (aminolevulinic acid) in conjunction with laser.					Patients also benefit from prolonged periods of non-treatment.
Ihenachor et al.[41] (USA, 2016)	Case report	N/Proc: 1/1 Age: 82 Gender: 1/0	Cyroptococcal laryngitis	NA	Disease Control	Complete resolution with combined therapy.	Office-based laser ablation is a viable treatment for cryptococcal lesions of the larynx that persist after medical therapy.
Centric et al.[25] (USA, 2014)	Retrospective uncontrolled (2005-2012)	N/Proc: 33/NA<br>Age: 49<br>Gender: 21/12	Vascular lesion 10 (31%)<br>RRP 8 (25%)<br>Granuloma 5 (16%)	NA	Complications Disease Control	26 (81%) patients had operative intervention other than in-office PDL therapy.<br>32 (97%) tolerated the procedure with no difficulty.<br>1 patient had anxiety shortly after the introduction of the telescope and did not tolerate in-office therapy at all.	For a selected group of patients with granulomatous or vascular lesions, in-office therapy with the PDL has proven safe and effective.

			Premalignant (dysplasia) 5 (16%)<br>Benign mass 2 (6%)<br>Anterior glottic web 1 (3%)<br>Amyloidosis 1 (3%)			There were no complications.	
Mortensen et al.[37]<br>(USA, 2008)	Prospective uncontrolled	N/Proc: 11/33<br>Age: 60.45<br>Gender: 8/3	Vocal fold scar	All patients had a power setting of 0.75 J.<br>Average distance of the laser from the site of the lesion is 4.29 mm.<br>Average number of pulses: 70.	Disease Control Voice Outcomes	VHI from 48.44 to 35.55 (P<.05) at 6 months posttreatment.<br>VHI functional from 14.73 to 11.11 (P <.05).<br>VHI physiological from 20.33 to 15.22 (P<.05).<br>VHI emotional from 14.0 to 9.22 (P<.05).	Serial PDL may be used in treating patients with established vocal fold scarring.

Kim et al.[26]<br><br>(South Korea, 2008)	Retrospective uncontrolled (2004-2006)	N/Proc: 62/63<br><br>Age: 44<br><br>Gender: 34/28	Vocal polyps	450 µs pulse width<br><br>750-1000 mJ per pulse output of 2 Hz<br><br>1-2 mm spot size, 2-45 J/cm2	Complications Disease Control Voice outcome	Complete regression of the vocal polyps in 60 patients. 95% regression in two patients. One patient with partial response required a second PDL surgery, after which there was complete remission. Mean follow-up 5.2 months.<br><br>GRBAS scale was significantly decreased after operation ($p<0.001$). The HNR (noise to harmonic ratio) increased, and jitter, shimmer, sPPQ (smoothed pitch perturbation quotient), sAPQ (smoothed amplitude perturbation quotient), and SPI (soft phonation index) decreased after the surgery ($P<0.05$)	The PDL resulted in precise, selective coagulation of the microvasculature without damage to the surrounding tissue.<br><br>PDL surgery appears to be safe and effective for office-based treatment of benign laryngeal disease and for all patients regardless of their overall medical condition.

Koufman et al.[32]<br><br>(USA, 2007)	Retrospective uncontrolled (2002-2006)	N/Proc:<br>- RRP: 59/212<br>- Leukoplakia and dysplasia: 25/79<br>- Granulomas cases: 23/40<br>- Reinke edema: 12/18<br><br>Age: 88<br><br>Gender: 113/38	RRP in the larynx or trachea<br><br>Glottal dysplasia/leukoplakia<br><br>Granuloma<br><br>Reinke edema (polypoid degeneration)	RRP: Median PDL power setting was 1.0 J (range 0.5-2.0 J). Mean N of pulses 236±240 (range 9-1887; median 161).<br><br>Leukoplakia and dysplasia: mean PDL power setting was 1.0 J (range 0.75-1.5 J). Mean N of pulses 117±68 (range 20-436; median 113).<br><br>Granulomas: mean power setting was 1.0 J (range 0.75-1.5 J). Mean N of pulses 137±85 (range 33-403; median 119).<br><br>Reinke's edema: mean power setting was 1.0 J (range 0.75-1.5 J). Mean N of pulses 89±55 (median 94.5, range 3-199)	Complications<br><br>Disease Control<br><br>Voice Outcome	RRP: mean of 3.6 procedures/patient (range 1-15). 15% (9/59) required subsequent OR. Mean follow-up 17 mo. No complications.<br><br>Leukoplakia and dysplasia: mean 3.2 procedures/patient (range 1-9). 5 patients required subsequent procedures in the OR. One patient developed vocal fold carcinoma. Mean follow-up 16 mo. There were no complications.<br><br>Granulomas: mean of 1.6 procedures/patient (range 1-5). Effective in resolving granulomas in 68% (13/19) of subjects. 6 subjects needed further surgery in the OR. Mean follow-up 12 mo. PDL fiber tip broke off in the trachea of one patient (immediately retrieved with a cup of forceps).<br><br>Reinke's edema: mean of 1.5 procedures/patient (range 1-3).	Bulky nonvascular lesions (e.g., exophytic papillomas or supraglottic cysts) are best excised/ablated with the CO2 or the Tm:YAG laser.<br><br>If hemostasis is a concern, the Tm:YAG is preferred, such as for large inflamed vocal process granulomas.<br><br>For leukoplakia, dysplasia, Reinke's edema, non-bulky papillomas, and especially papillomas at the anterior commissure, the PDL is preferred.

						Resolution in all cases. Mean follow-up 7.3 mo. 2 subjects had vocal fold hemorrhages.	

Ivey et al.[27]<br><br>(USA, 2007)	Prospective uncontrolled (2006)	N/Proc: 29/33<br><br>Age: NA<br><br>Gender: NA	Vascular polyps	450 μs pulse duration and 1-second interpulse duration, 0.75 J	Complications<br><br>Disease Control<br><br>Voice outcome	4 patients reported smelling smoke during the procedure. 1 patient had some flushing attributed to the anesthetic. All patients were able to finish their treatment sessions under local anesthesia.<br><br>38% of cases improved by at least 70%.<br><br>48% had lesions that improved by 50%.<br><br>8 patients need a second intervention.	Acoustic measurements and Voice Handicap Index testing were not routinely performed before and after PDL treatment.<br><br>No correlation between the energy delivered and the amount of polyp resolution.<br><br>Lesions occupying less than one-third of the vocal fold seem to have the most effective clearing with this type of laser.

Franco[33] (USA, 2007)	Prospective uncontrolled (2001-2006)	N/Proc: 12/28<br>- 16 with ALA<br>- 12 without ALA.<br>- 18 unsedated in-office<br><br>Age: 63.5<br>Gender: 12/0	Keratosis/ atypia	1.5 to 3.0 cc of 20% ALA aerosolized and sprayed into the larynx 1 to 3 hours before the surgery and activated with a PhotoGenica SV 585-PDL	Complications<br>Disease Control	4 follow-up lost.<br>78% reduction (range 10-100%) in the amount of keratosis within each patient. 6 (75%) patients had ≥85% reduction in keratosis.<br>12 complained of mild nausea that lasted up to 3 hours when the ALA was sprayed while awake. This did not preclude finishing those procedures. There were no anaphylactic reactions, no reports of generalized photosensitivity, unexpected persistent dysphonia, or dysphagia after ALA treatment.	No statistical differences between the procedures performed in the operating room compared with those in the outpatient awake setting with respect to the number of pulses, the energy of the laser used, or the interval between cases.

Mouadeb et al.[28]<br><br>(USA, 2007)	Retrospective uncontrolled (2004-2006)	N/Proc: 47/113<br><br>Age: 63.5<br><br>Gender: 12/0	RRP 70 (60%)<br><br>Reinke's edema 16 (13%)<br><br>Leukoplakia 13 (11%)<br><br>Polyp 8 (7%)<br><br>Granuloma 8 (7%)<br><br>Varices 1 (1%)<br><br>Vallecular cyst 1 (1%)	450 µs pulse width, 0.75 to 1.5 J per pulse output, 2 Hz repetition rate, 0.6 mm fiber, approximately 1 to 2 mm laser spot size, 65-250 J/cm2 fluence.	Complications Disease Control Voice outcome	Mean follow-up 13.5 mo.<br><br>RRP:<br><br>- Mean of 3.5 procedures<br>- 104 (89%) ablation of all visible disease<br>- 13 (11%) treatments required early termination (e.g., inability to achieve a comfortable level of anesthesia, inadequate visualization, and edema<br>- 15 (32%) patients required at least one surgical procedure under general anesthesia<br><br>1 patient with Reinke's edema developed postprocedure stridor that required a 3-day hospital admission for observation and intravenous corticosteroids.<br><br>No incidence of vocal fold webs or scarring, and no other complications occurred.	Use of the PDL for unsedated office laryngeal surgery is safe and effective.<br><br>Patients generally prefer an unsedated office procedure.<br><br>The most frequent use for PDL treatment at a tertiary referral center appears to be RRP.<br><br>The risk of web formation at the anterior commissure scar is low.<br><br>The threshold for recommending surgical intervention is lowered with the use of the PDL.

						The mean VHI improved from a preoperative value of 21±10 to a postoperative value of 14±11 (P<0.001).	

Rees et al.[34] (USA, 2006)	Retrospective uncontrolled (2002-2005)	N/Proc: 131/328<br>Age: 63.5<br>Gender: 12/0	RRP 172 (52%)<br>Leukoplakia 62 (19%)<br>Granuloma 27 (8%)	NA	Pain and comfort	54 (61%) subjects have undergone previous treatment(s) under general anesthesia. 47 (87%) of these 54 subjects stated they preferred the in-office unsedated PDL treatments to the surgeries under general anesthesia.<br>75 (84%) did not use any pain medicines.	Office-based PDL treatment is well-tolerated.<br>Most patients who have undergone previous surgery in the operating room feel the PDL procedures are more comfortable, safer, and more convenient. These patients prefer PDL procedures in the clinic over procedures in the operating room.

Clyne et al.[36]<br><br>(USA, 2005)	Retrospective uncontrolled (2002-2003)	N/Proc: 10/>10 Age: 58 Gender: NA	Granulomas	450 Jls pulse width, 1 J per pulse maximum output of 1 Hz, 1 to 2 mm spot size, 38 to 255 J/cm2 fluence	Complications Disease Control	5 of the 10 patients had resolution of their lesions, and 3 had a partial response. Two were unchanged.<br><br>Average follow-up 6 months<br><br>No complications.	The current study involved patients in whom antireflux therapy and voice therapy failed.<br><br>PDL treatment in conjunction with medical and behavioral management, rather than reserving it for cases refractory to medical and behavioral management.

Zeitels et al.[35]<br><br>(USA, 2004)	Prospective uncontrolled (2001-2002)	N/Proc: 51/82<br><br>Age: NA<br><br>Gender: NA	RRP 17<br>Dysplasia 34	450 μs pulse width, 2.0 J per-pulse maximum output, 2 Hz repetition rate, 0.6 mm fiber, approximately 1 to 2 mm spot size, 65 to 250 J/cm2 fluence	Complications<br><br>Disease Control<br><br>Voice Outcome	5 cases were aborted, 2 because of inadequate exposure and 3 because of discomfort.<br><br>68 (88%) have >50% disease regression. 9 (12%) have a 25% to 50% disease regression.<br><br>34 cases, patients reported that their voices were improved.<br><br>2 mild episodes of epistaxis.	Not observe that the PDL alters the natural history of recurrence of papillomatosis or dysplasia.
